# Electromigration-induced formation of percolating adsorbate islands during condensation from the gaseous phase: a computational study

**DOI:** 10.3762/bjnano.12.55

**Published:** 2021-07-13

**Authors:** Alina V Dvornichenko, Vasyl O Kharchenko, Dmitrii O Kharchenko

**Affiliations:** 1 Sumy State University, 2 Rimskii-Korsakov St., 40007 Sumy, Ukraine; 2 Institute of Applied Physics, National Academy of Sciences of Ukraine, 58 Petropavlivska St., 40000 Sumy, Ukraine

**Keywords:** adsorptive systems, electromigration, numerical simulations, pattern formation, thin films

## Abstract

We provide a computational study of a change in the morphology of a growing thin film during condensation caused by electromigration effects. It will be shown, that separated circular adsorbate islands, realized in an isotropic system, become elongated in the direction of the applied electrical field. We discuss the dependence of the critical value of the strength of the applied electrical field, responsible for the formation of percolating adsorbate islands, on main control parameters. This study provides insight into details of electromigration effects during the self-organization of adatoms into percolating adsorbate islands during condensation from the gaseous phase. We will show that the elongated morphology of adsorbate islands remains stable if the electric field is turned off.

## Introduction

The processes of surface pattern formation at elevated temperatures are the result of the self-organization of adsorbed atoms. They are usually governed by kinetic factors, that is, the rates of processes realized on the growing surface. One of the interesting phenomena influencing the formation of surface morphology at high temperatures is electromigration, which occurs when an electric current is applied to the substrate [1–2]. Electromigration (EM) is the movement of atoms caused by an electric current. It occurs due to the scattering of conduction electrons on atoms that move in a solid due to diffusion processes. The electron wind force, which arises during the transmission of the momentum by the conduction electrons, pushes the ions in the direction of the electron flow. As a result, the diffusion processes in the material become anisotropic and directed. This results in the exchange of atoms and the neighboring vacancies in the direction of electron flow leading to an accumulation of atoms at the anode and vacancies at the cathode [1–2].

Current trends in computer technology, namely, reducing the size of integrated circuits, increasing their power, and increasing the density of elements, have led to an increase in current density and, consequently, to a greater manifestation of EM. It is recognized as one of the main problems in the reliability of polar connections in microelectronics. The most common failure of electronics devices is the rupture of the electrical circuit caused by pores induced by the growth of intermetallic compounds between the solder and the metal contact of the integrated circuit (flip-chip technology) [3–7].

The vast majority of research on EM began in the 1970s. Such studies were mostly conducted experimentally. It was shown that the influence of the electric current applied to the substrate leads to efficient surface heating and, as a consequence, the induced spatially directed drift of adatoms begins to play a significant role in the formation of surface structures during deposition [8–11]. Reorganization of the step structure of the adsorbate islands was observed on silicon substrates [12–13]. Strong effects of EM were manifested in the processes of evolution of vanadium surface morphology [14], and in the epitaxial growth of semiconductor heterostructures [15]. It was found that at low deposition temperatures the growth of surface structures occurs according to the Stransky–Krastanov growth regime [16–19], whereas at elevated temperatures such processes are associated with the solid dissolution of the layers of precipitated material [20–21]. Surface EM [22–23] is widely used in the study of thin solid films, for example, for the formation of nanocontacts [24–25], the direction of movement of monoatomic steps and islands on the surface of crystals [22–23,26], the formation of faces on the surface of crystals and crystalline surfaces [27]. Studies of the effects induced by surface EM in thin monocomponent monocrystalline films include the formation of surface steps [28–31], faceting of the surface [32–37], elimination of instability of surface morphology caused by stress and wetting of the substrate [38–41], the evolution of contact irregularities in switches of microelectromechanical systems [42], control of surface roughness [43] and morphology of islands or nanowires [26,44–47], as well as control of adsorbate transfer to graphene [48]. Thus, the effects of EM induced by the presence of a potential difference on opposite sides of the substrate can significantly affect the dynamics of the evolution of surface morphology at elevated temperatures. This effect can lead to a change in the morphology of the surface compared to the isotropic case of deposition without the presence of an external field.

Mathematical and numerical modeling of nanostructured thin film growth processes allows one to analyze in detail the dynamics of this process, to establish the influence of basic factors (pressure inside the chamber, deposition temperature, energy characteristics, and external influence) on the morphology of the growing surface and type and size of surface structures. One of the most common approaches for mathematical modeling of these processes is based on the reaction–diffusion models [49–61]. This approach generally allows one to make certain recommendations for adjusting the technological conditions for growing thin films with specified physical and chemical properties.

In this article we perform a computational study of a change in the morphology of a growing thin film during condensation induced by electromigration effects and show that separated circular adsorbate islands realized in the isotropic system become elongated in the direction of the applied electrical field. In order to construct the isotropic model of the adsorptive gas–condensate system we will proceed in a manner similar to our previous works [50,59,62–64]. The novelty of the present work lies in the introduction of the anisotropic surface current of adsorbate induced by the applied electrical field and in the determination of its influence on the morphology of the growing thin film.

The paper is organized in the following way. In the section “Model” we derive the one-layer model of reaction–diffusion type for the spatio-temporal evolution of adsorbate concentration on the substrate. After that, we discuss the results of numerical simulations. The main conclusions are collected in the last section.

## Model

In order to describe the evolution of the adsorbate concentration on the first growing layer of the multilayer system during condensation from the gaseous phase, let us consider a model with only one type of particles. On the mesoscopic level of description it is convenient to divide the surface of the substrate into cells with a linear size 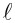 and consider the local concentration *x* of adsorbate in a cell as the ratio between the number of adsorbed particles (adatoms) in the cell and the total number of places for adsorption (nodes) in each cell. In this case, the concentration of adsorbate in each cell is *x*(**r**,*t*) ∈ [0,1], where *t* is the time variable and **r** is the spatial coordinate. The spatio-temporal evolution of the adsorbate concentration field on the first growing layer is given by the equation of the reaction–diffusion type in the following form:

[1] $$\frac{\partial x\left(r,t\right)}{\partial t}=R\left(x\right)-\nabla \cdot J+\xi \left(r,t\right),$$

where *R*(*x*) is responsible for the reaction component, describing quasichemical reactions (birth–death processes), **J** is the surface flow of the adsorbate on the substrate associated with mass transfer; ∇ ≡ ∂/∂**r**. For simplicity, we assume, that there are no cross-effects in mass transfer, and that Fick’s or Fourier’s laws adequately describe mass transport. The last term in Equation 1 represents a stochastic source, which in the simplest case is chosen as white zero-mean delta-correlated Gaussian noise: ⟨ξ(**r**,*t*)⟩ = 0, ⟨ξ(**r**,*t*)ξ(**r’**,*t*’)⟩ = σ^2^δ(**r**− **r’**)(*t* − *t*’); σ^2^ is the intensity of fluctuations. This source takes into account the effects of redistribution of adatoms at the microscopic level, describing the system at the mesoscopic level.

### Reaction term

During condensation processes atoms from the gaseous phase attach to the substrate and become adatoms. The adsorption rate *k*_a_ = 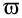
*p* exp(−*E*_ads_/*T*) is defined by the pressure of the gaseous phase *p*, activation energy for adsorption *E*_ads_, and the frequency factor 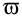; *T* is the temperature measured in units of energy (eV). Adatoms can desorb back into a gaseous phase with the rate *k*_d0_ = 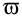
exp(−*E*_des_/*T*); *E*_des_ is the activation energy for desorption. The desorption rate *k*_d0_ defines the average lifetime of the adatom on the layer τ_d_ in the common way: τ_d_ = [*k*_d0_]^−1^. By considering adatoms as interacting mobile particles, the desorption rate is corrected by taking into account the effects of the interaction of adatoms with the potential *U*(**r**). In this case, the desorption rate of the interacting particles takes the general form *k*_d_ = *k*_d0_exp(*U*/*T*). It was shown previously, that the system with adsorption and desorption reactions only is stable to any spatial instability [53–58]. The transient patterns realized during system evolution will disappear at large time scales leading to the homogeneous distribution of the coverage field [59]. In order to stabilize these transient patterns the reaction component *R*(*x*) can be generalized by introducing the term responsible for the non-equilibrium reactions describing associative desorption, or formation of stable complexes [53–60,65]. In a real situation, atoms from the gaseous phase can be adsorbed not only on the substrate but also on the adatoms of the first layer leading to a growth of multilayer adsorbate islands due to pairwise attractive interaction between adatoms. In such a case the reaction term *R*(*x*) should take into account the term *f*_t_ responsible for the transitions of adatoms between neighboring layers, representing vertical diffusion. These transitions require free sites on the target layer. For the adsorbate concentration on the first growing layer for *f_t_* one gets: *f_t_* = *k*_↓_*y*(1 − *x*) − *k*_↑_*x*(1 − *y*), where *y*(**r**,*t*) is the adsorbate concentration on the second layer; *k*_↑_*_,_*_↓_ are the rates of bottom-up motion and vice versa, respectively. Next we assume, that the rates of vertical motion of adatoms are equivalent, *k*_↑_ = *k*_↓_, giving *f_t_* = *k*_↕_(*y* − *x*). By using the approach proposed in [62–63] one can express the adsorbate concentration on the second layer through the one on the first layer in the following form: *y* = (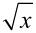 − β/2)^2^, where β is defined as a ratio between the terrace width of the multilayer pyramidal adsorbate islands and the linear size of the system.

### Lateral flux

The lateral adsorbate flow on the first layer includes both free surface diffusion **J_0_**, defined according to Fick’s law **J_0_** = −*D*_↔_∇*x* with a lateral diffusion coefficient *D*_↔_, and a diffusion component **J_int_** defined by the interaction potential of the adsorbate *U*(**r**) as follows: **J_int_** = (*D*_↔_/*T*)μ(*x*)∇*U*, where the kinetic coefficient μ(*x*) = *x*(1 − *x*) determines that this diffusion is possible only on sites free of adsorbate. The interaction potential *U*(**r**) can be defined in the framework of the self-consistent approximation through the binary attraction potential for two adatoms *u*(**r**) separated by the distance |**r**|, in the form *U*(**r**) = 
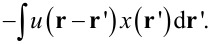
 Assuming that the binary potential *u*(**r**) has a symmetrical shape, that is, 
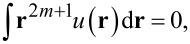
 *m* = 1, …, ∞, we choose for it a Gaussian in the standard form:

[2]
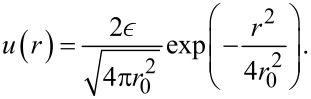


Here 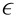 and *r*_0_ are the interaction energy of the adsorbate and the interaction radius, respectively. By assuming that the concentration of adsorbate *x* does not crucially change within the interaction radius *r*_0_, we can use an expansion:

[3]
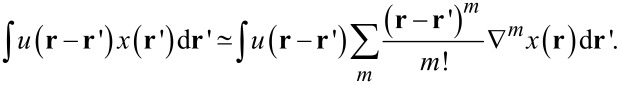


By substituting Equation 2 into Equation 3 with the condition 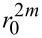 → 0 at *m >* 2, the interaction potential of the adsorbate *U*(**r**) becomes the following form: 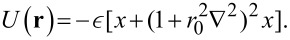 This self-consistent approach was widely used not only in the study of the formation of nanoscale structures of adsorbate by condensation [49–63] and epitaxy [66–68], but also in the study of the formation of nanoscale clusters of point defects in solids exposed to irradiating sources [69–73].

### Electromigration effects

**Figure 1:**
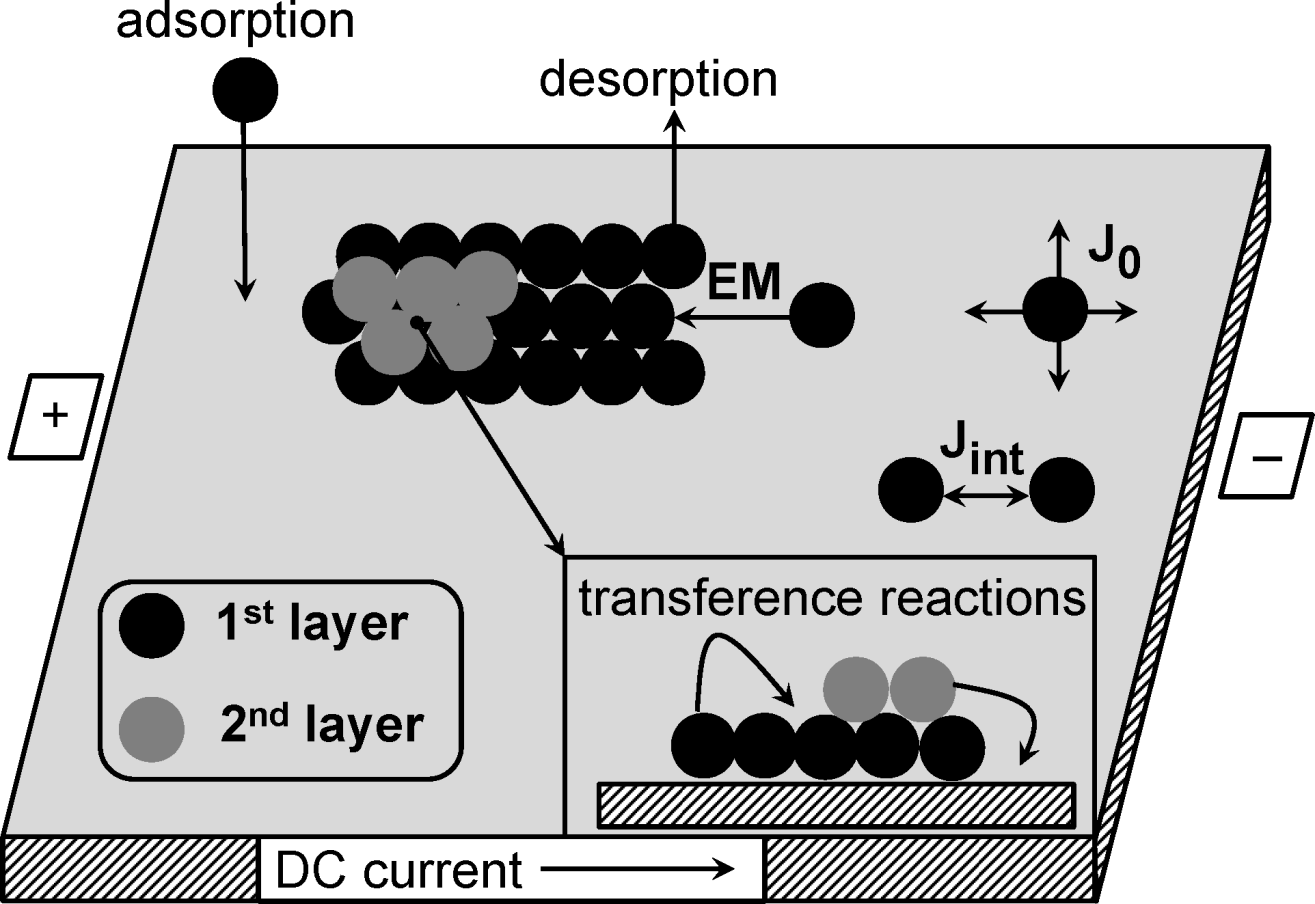
A schematic view of the two-layers model. Effects of upper layers are ignored in the model.

The main purpose of this work is to study the effect of electromigration on the dynamics of surface growth during deposition, its morphology, and statistical properties. The electric field applied to a substrate with a direction parallel to the substrate results in a change in the internal local electric field leading to a directed force **F_el_** = *eZ***E**. The strength |**E**| = −Φ/*L* is determined by the potential difference Φ and the linear size of the substrate *L* (distance between anode and cathode); *e* is the electron charge. The direction of the force **F_el_** is defined by the effective valence *Z*, which is negative for most metals. Thus, the adsorbed atoms move in the opposite direction to the electric field. In the general case if the electric field is applied across the substrate, then the electrical conductance will vary as the thickness of the system will vary along the direction of the applied electric field for a multilayer thick absorbate islands on a substrate system. There will be current crowding at the base of the islands and the electric field needs to be computed by solving the appropriate electrostatic boundary value problem [74]. In the present study we aimed to analyze a change in the morphology of the thin film on a substrate (first growing layer), assuming that upper layers will repeat the morphology of the first one. In this connection, we simplify our model by neglecting effects realized in multilayer thick absorbate islands on a substrate. In order to take into account the effects of electromigration we assume that the surface diffusion of adatoms on the substrate is accompanied by the directional motion of adatoms in one of the directions induced by an electric field: ±*k*_em_∇*_x_x*(**r**), where it is taken into account that the electric field is directed along the direction *x*, and the sign ± is determined by the relative position of the cathode and anode. The rate of directed motion *k*_em_ is proportional to the strength **E** of the electric field. A schematic view of the model is shown in Figure 1.

### Dimensionless system

Next, it is more convenient to move to dimensionless constants α = *k*_a_/*k*_d0_, *k*_∥_ = *k*_↕_/*k*_d0_, *D*_em_ = *k*_em_/*k*_d0_ by scaling time in units of lifetime of adatoms τ_d_ and the spatial coordinate in units of diffusion length 
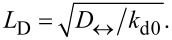
 The spatio-temporal evolution of adsorbate concentration on the first growing layer during condensation is described by the following reaction–diffusion system:

[4] $$\partial_{\text{t}}x=R\left(x\right)-\nabla \cdot J_{tot}+D_{\text{em}}\nabla_{x}x+\xi \left(r,t\right),$$

where the reaction term *R*(*x*) has the form

[5]
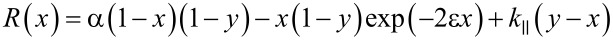


and the total diffusion flux **J_tot_** reads

[6] $$J_{tot}=-\nabla x+\varepsilon \mu \left(x\right)\nabla \cdot \left[x+{\left(1+\rho^{2}\nabla^{2}\right)}^{2}x\right].$$

In the reaction part we take into account that adsorption is possible on free sites on the current layer and requires free sites on the next layer (first term in Equation 5), and that desorption requires free sites on the next layer (second term in Equation 5). In Equation 5 and Equation 6 we use ε = 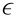/*T* and introduce the spatial scale ρ ≡ *r*_0_/*L*_D_. The estimation for the interaction radius *r*_0_ and diffusion length gives *r*_0_ ≈ 10^−9^ m and *L*_D_ ≈ 10^−7^ m [55–56]. Hence, for the system under consideration one has *r*_0_ ≪ *L*_D_. Therefore, without loss of generality, next, we use the limit ρ^4^ → 0 in Equation 6. In further study we fix β = 0.1 and σ^2^ = 0.01.

## Results and Discussion

### Stability analysis

In order to determine an influence of the electromigration effects characterized by the electrical field strength *D*_em_ onto surface morphology, first, we will define the ranges of system parameters in which the surface patterns, realized on a surface during deposition, will be stable. To this end we exploit standard linear stability analysis in order to characterize the stability of the stationary homogeneous state *x*_st_ against inhomogeneous perturbations. In this approach the state *x*_st_ is defined from the relation ∂*_t_x* = ∇*x* = 0, giving *R*(*x*_st_) = 0. The deviation δ*x* from the stationary state δ*x* = *x* − *x*_st_ can be found in a standard form: δ*x* ∝ *e*^λ(^*^k^*^)^*^t^e^ikr^*, where *k* is the wave number and λ(*k*) is the stability exponent. In order to linearize the reaction term *R*(*x*) we expand it in the vicinity of *x*_st_ up to a term of the first order for δ*x*, which gives 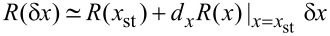. In such a case, from Equation 4 for the deviation δ*x* we get the expression for the stability exponent λ(*k*) in the form:

[7] $$\lambda \left(k\right)=\lambda_{0}-\kappa^{2}\left[1-2\varepsilon \mu \left(x_{\text{st}}\right)\left(1-\rho^{2}\kappa^{2}\right)\right].$$

where the notation 
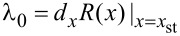
 is used and the reduced wave number κ = *kL*_D_ is introduced. By analyzing the dependence of the stability exponent λ(κ) on the control parameters, namely, adsorption rate α, interaction strength ε, and the rate of vertical motion of adatoms *k*_∥_, one can define domains of main system parameters, in which pattern formation is possible. In particular, for the case λ(κ) ≤ 0 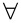
κ any spatial instability that can be realized at the initial stages of the system evolution disappears at long time scales leading to a homogeneous distribution of the field *x*(*r*). For the considered problem this means that the adsorbate will cover the substrate homogeneously and no stationary patterns are possible. For a special choice of control parameters one can get λ(κ) *>* 0 at κ ∈ [κ_1_,κ_2_] with κ_1,2_ ≠ 0. In this case the spatial modulations will result in a stationary spatial configuration of the field *x*(*r*) with high- and low-density states, leading to pattern formation. For the system under consideration these conditions are responsible for the formation of separated adsorbate clusters (nanodots on the substrate) or separated holes in the adsorbate matrix. This approach was previously used in problems of pattern formation in different types of reaction–diffusion systems (see, for example, [75–77]).

**Figure 2:**
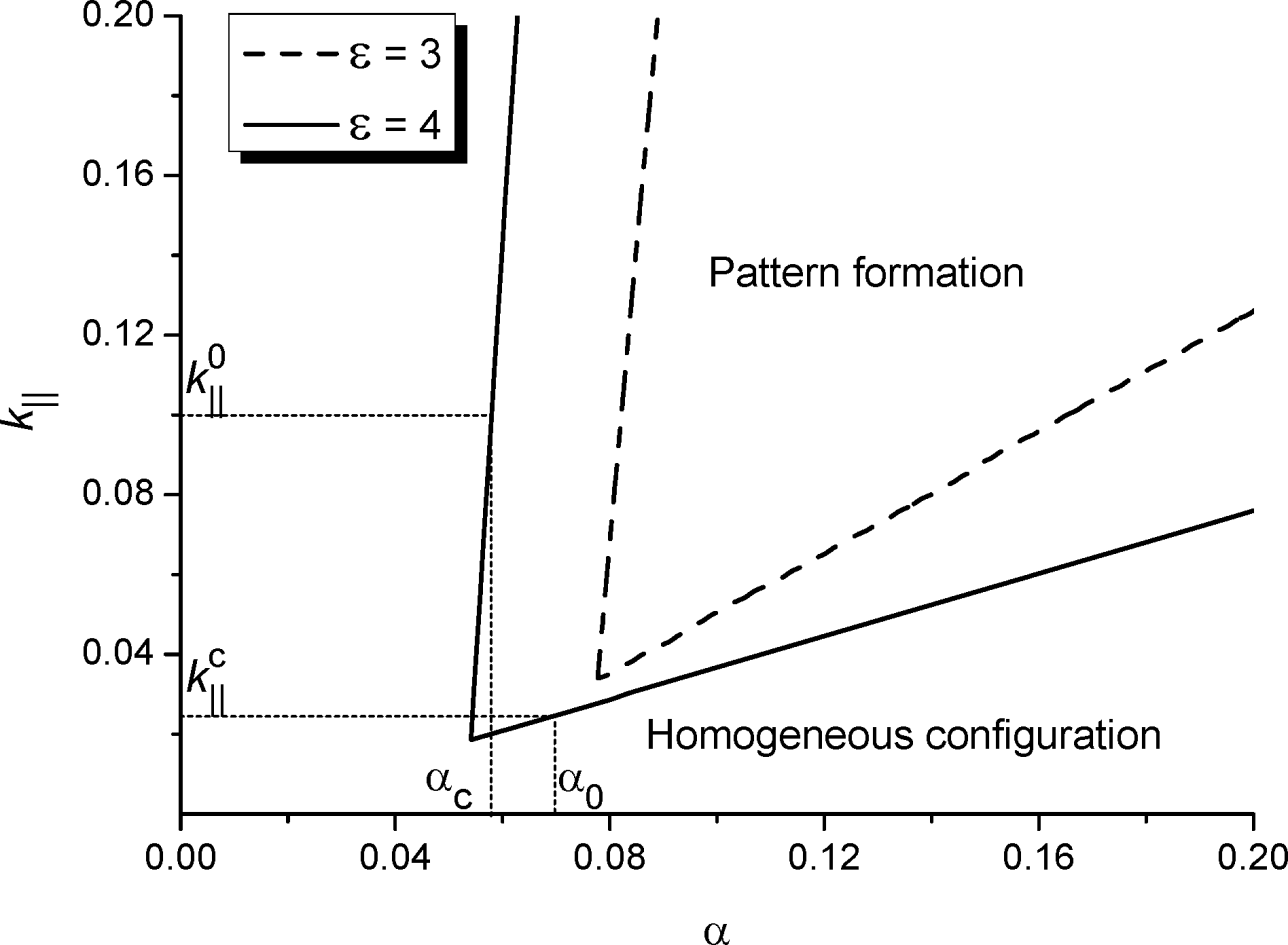
Stability diagram *k*_∥_(α) at different values of the interaction strength ε.

By varying the adsorption rate α and the rate of adatom transference between layers *k*_∥_ we have obtained a stability diagram, shown in Figure 2 for different values of the interaction strength ε. Here, outside the cusp bounded by solid or dash lines, the adsorbate will homogeneously cover the substrate and no surface patterns will realize. Inside the bounded domain during deposition spatial instabilities will promote pattern formation processes, that is, separated adsorbate structures on a substrate or separated holes inside the adsorbate matrix. It follows that in the case *k*_∥_ → 0 (no mass transfer between layers) or small α adsorption/desorption processes can not induce pattern formation. For a fixed value *k*_∥_ = 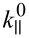 an increase of α leads to ordering processes at α ≥ α_c_. At a fixed value of the adsorption coefficient α = α_0_ adsorbates will self-organize into surface structures if the rate of transference reactions *k*_∥_ exceeds the critical value 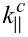. It is seen from Figure 2 that an increase in the values of the interaction strength ε extends the domain of α and *k*_∥_ in which patterning is possible.

### Numerical simulations

**Figure 3:**
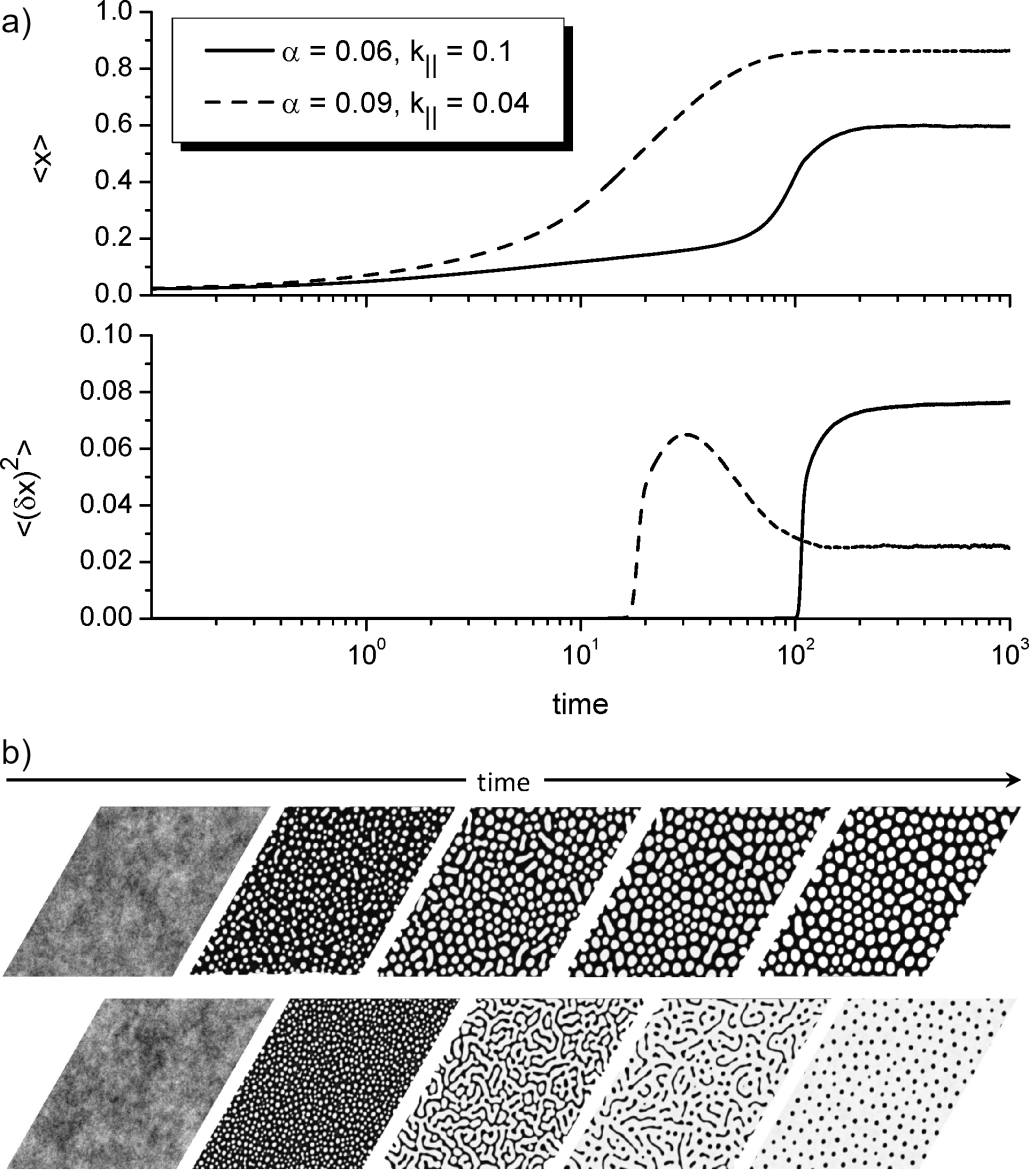
Evolution of the mean adsorbate concentration ⟨*x*⟩ (top panel in Figure 3a) and the dispersion ⟨(δ*x*)^2^⟩ (bottom panel in Figure 3a). Figure 3b shows snapshots of the system evolution for two different cases, that is, close to left border of the domain of pattern formation at α = 0.06 and *k*_∥_ = 0.1 (top panel) and close to right border of the domain of pattern formation at α = 0.09, *k*_∥_ = 0.04 (bottom panel). The adsorbate concentration on the substrate is shown in the gray scale from black for *x* = 0 to white for *x* = 1.

In order to perform numerical simulations of the process of pattern formation during deposition we will proceed in a manner closely related to [63–64]. We will solve numerically Equation 4 on a two-dimensional hexagonal grid of the linear size *L* = *N*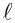 with *N* = 512; 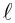 = 0.5 is the spatial integration step; Δ*t* = 10^−4^ is the time step. By using a relation *L*_D_/*r*_0_ = 40 for the linear size of the computational grid *L* we get *L* = 25.6*L*_D_. As initial conditions for the coverage field we use *x*(**r**,0) = 0, assuming that the substrate is free of adatoms; the boundary conditions are periodic. In all simulations we put ε = 4.0. Let us discuss the dynamics of pattern formation inside the cusp, shown in Figure 3. Here, in Figure 3a we present temporal evolution of both the mean adsorbate concentration on the substrate ⟨*x*⟩ (top panel) and the dispersion ⟨(δ*x*)^2^⟩ (bottom panel). The dispersion ⟨(δ*x*)^2^⟩ = ⟨*x*^2^⟩ − ⟨*x*⟩^2^ is an order parameter for pattern formation. If ⟨(δ*x*)^2^⟩ ≃ 0 then the field *x*(**r**) is homogeneously distributed and no patterns are possible. The growing dynamics ⟨(δ*x*)^2^⟩(*t*) indicates ordering of the field *x*(**r**) with formation of dense (*x* → 1) and diluted (*x* → 0) phases. In the case ⟨(δ*x*)^2^⟩(*t*) = const *>* 0 the spatial configuration becomes stable meaning formation of stationary surface patterns. In Figure 3b we show snapshots of the system evolution for two different cases, that is, close to the left border of the domain of pattern formation at α = 0.06 and *k*_∥_ = 0.1 (top panel) and close to the right border of the domain of pattern formation at α = 0.09 and *k*_∥_ = 0.04 (bottom panel). Here the adsorbate concentration on the substrate is shown in the gray scale from black for *x* = 0 to white for *x* = 1.

From Figure 3a it follows that during the initial stages of the condensation process the mean adsorbate concentration ⟨*x*⟩ increases, and after the transient regime it attains a constant value, which depends on the system parameters (see top panel in Figure 3a). The dispersion ⟨(δ*x*)^2^⟩ takes values close to zero at the initial stage meaning a quasihomogeneous distribution of adsorbate on the substrate (see the bottom panel in Figure 3a and the first snapshots in Figure 3b). After the incubation period dispersion starts to grow fast, leading to the formation of small adsorbate islands on a substrate (see the second snapshots in Figure 3b). These islands diffuse and interact. At large time scales the dispersion attains a constant non-zero value and the surface layer is characterized by stationary surface patterns, that is, separated adsorbate islands on the substrate (top panel in Figure 3b) or separated holes inside the adsorbate matrix (bottom panel in Figure 3b). It follows, that a variation in the adsorption rate α and/or the rate of the transference reactions *k*_∥_ can lead to different morphology of the surface layer.

**Figure 4:**
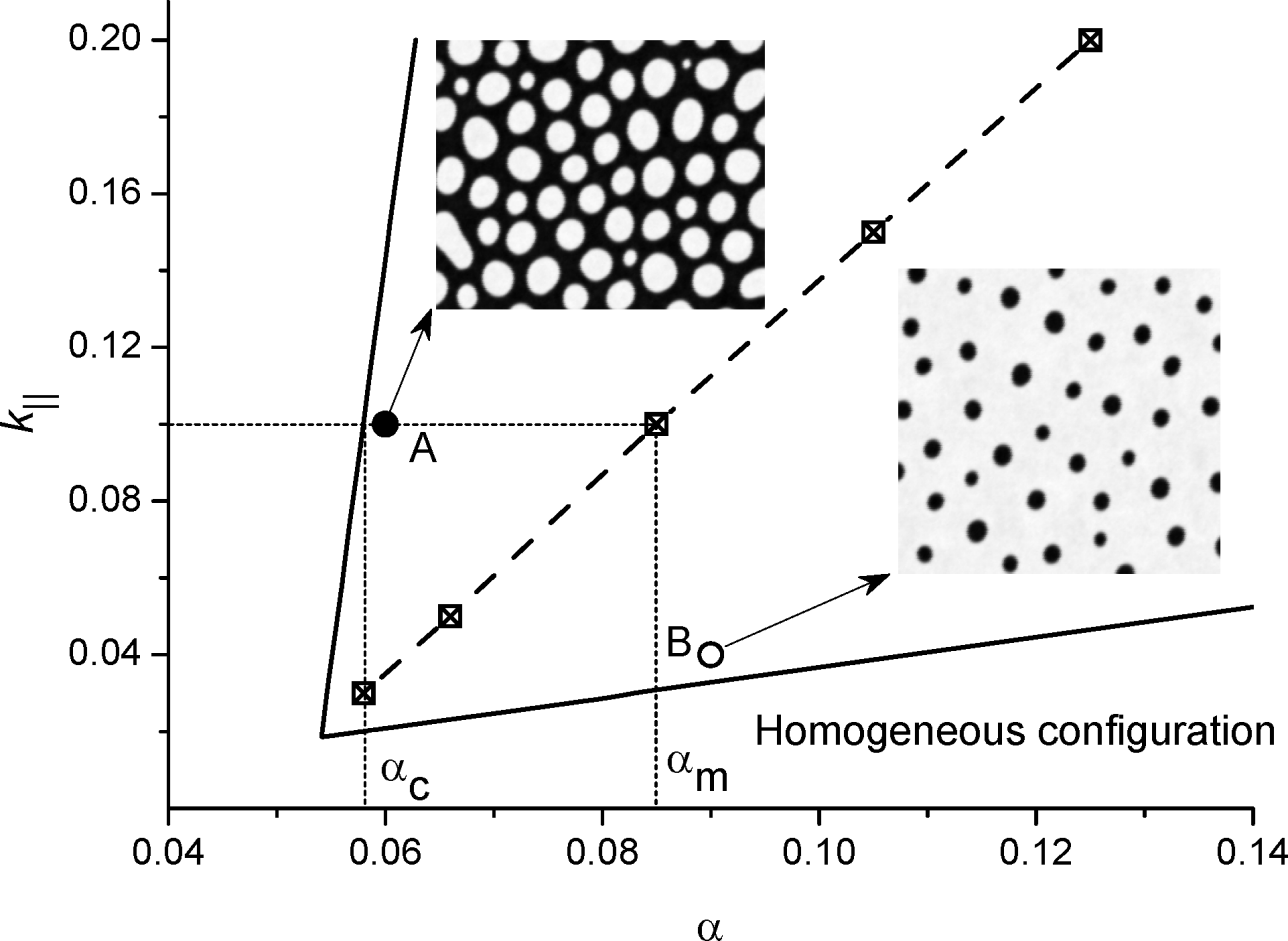
Stability diagram *k*_∥_(α) at ε = 4 and typical patterns in different domains: separated adsorbate islands for α = 0.06 (up inset) and *k*_∥_ = 0.1 and separated holes in adsorbate matrix for α = 0.09, *k*_∥_ = 0.04 (bottom inset).

In Figure 4 we show the stability diagram at ε = 4 and the dependence α*_m_*(*k*_∥_), which was obtained in the framework of numerical simulations for which percolating clusters of adsorbate are realized, by squares and the dashed line. Insets correspond to typical patterns in different domains of the stability diagram. Hence, an increase in an adsorption rate from the critical value α_c_(ε,β) leads to morphological transformation from separated adsorbate islands on the substrate towards separated holes inside the adsorbate matrix at α = α_m_. An increase in the rate of adatoms transference between layers, *k*_∥_, acts oppositely.

**Figure 5:**
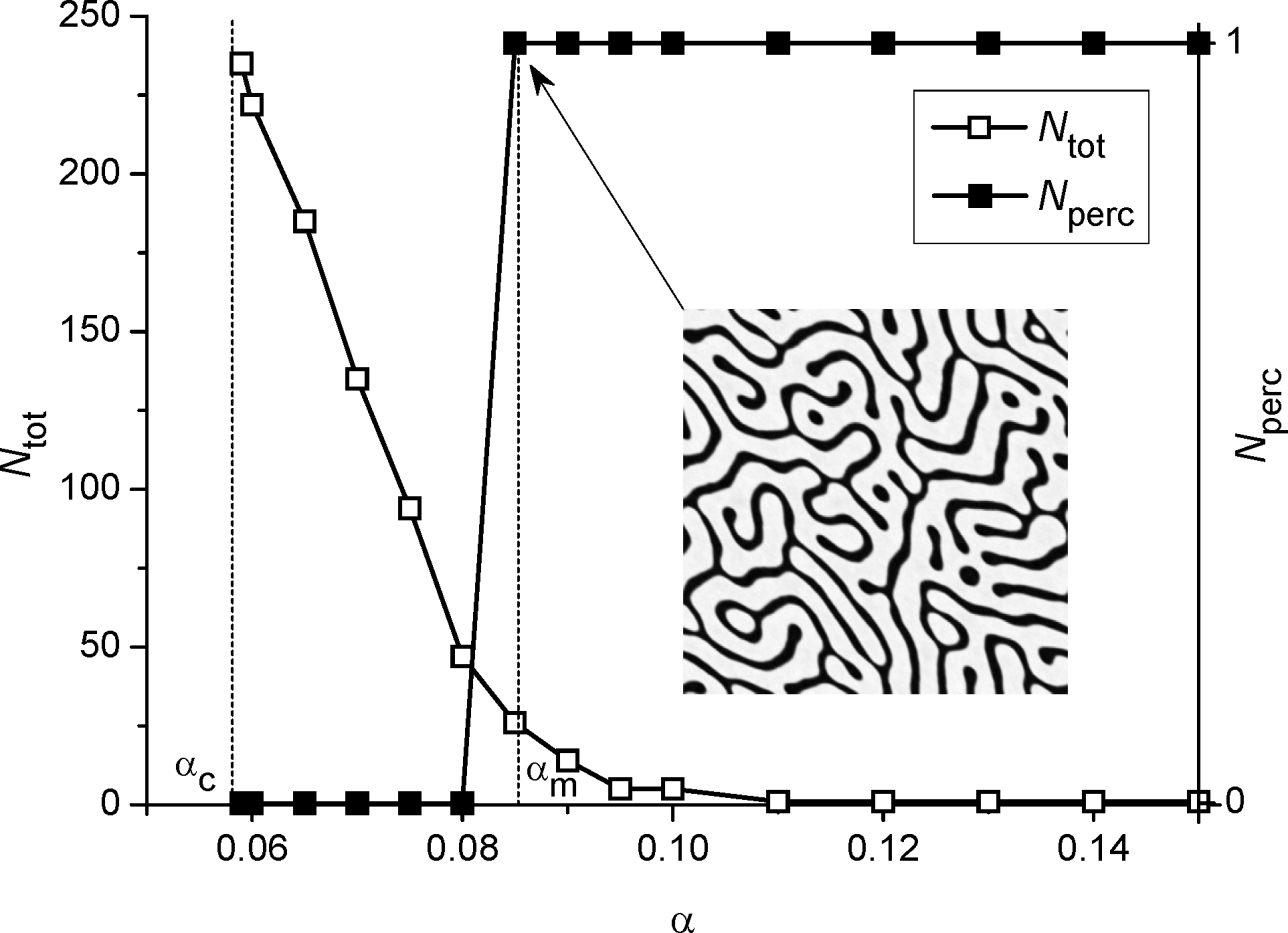
Dependencies of the total amount of adsorbate clusters *N*_tot_ and number of percolating clusters *N*_perc_ on the adsorption coefficient α at *k*_∥_ = 0.1 and *D*_em_ = 0.

In Figure 5 we show dependencies of the total amount of adsorbate clusters *N*_tot_ and number of percolating clusters *N*_perc_ on the adsorption coefficient α at *k*_∥_ = 0.1. It follows, that starting from α = α_c_ the total amount of adsorbate islands *N*_tot_ decreases with adsorption coefficient α to *N*_tot_ = 1 (see empty squares in Figure 5). At α *<* α_m_ there are no percolating islands of adsorbate (*N*_perc_ = 0); at α ≥ α_m_ one has *N*_perc_ = 1 independent on α (see filled squares in Figure 5).

**Figure 6:**
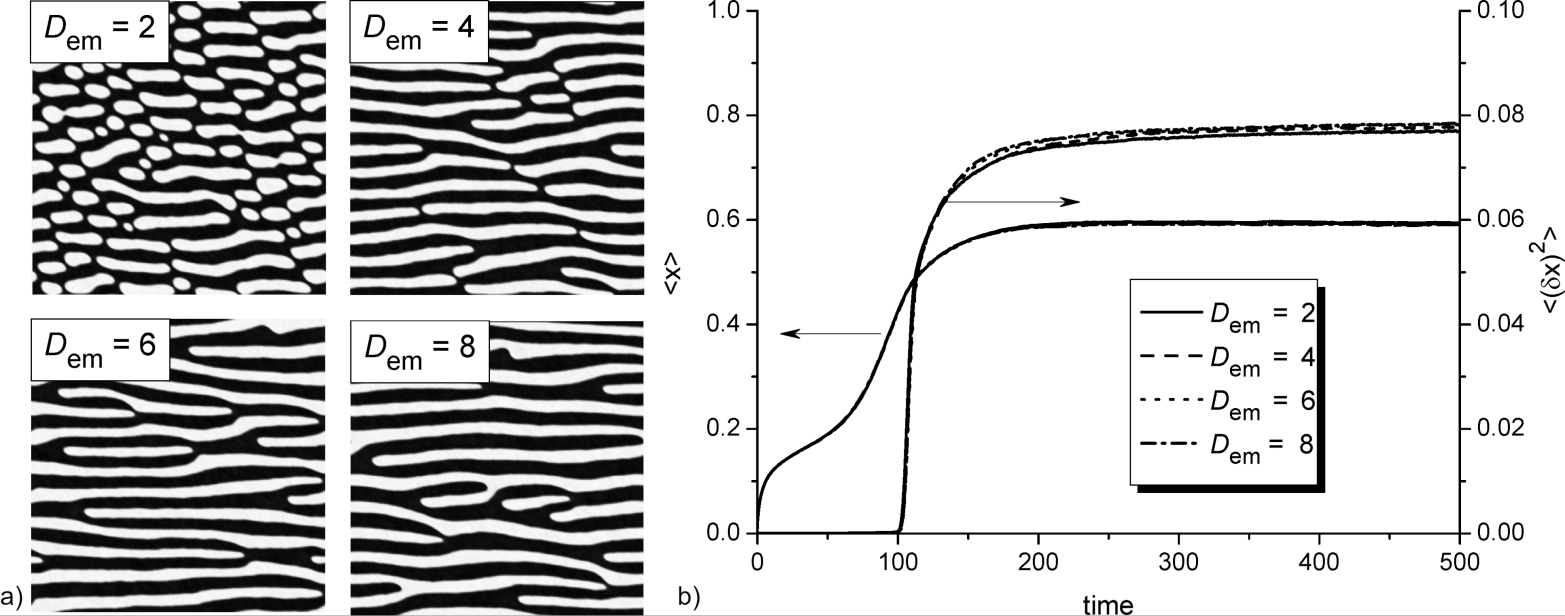
Typical snapshots of the quasistationary surface patterns for different values of the electrical field strength *D*_em_ at *k*_∥_ = 0.1 and α = 0.06 (a) and evolution of the mean adsorbate concentration ⟨*x*⟩ and the dispersion ⟨(δ*x*)^2^⟩ (b).

Next, we will discuss the influence of the electromigration effects on a change in surface morphology. To that end we fix α = 0.06 and *k*_∥_ = 0.1, at which in an isotropic system separated adsorbate islands are formed (see top snapshot in Figure 4). Typical snapshots of the quasistationary surface patterns (at *t* = 500) for different values of the electrical field strength *D*_em_ are shown in Figure 6a. It follows, that the applied electrical field with small strength provides the formation of elongated patterns in the field direction (see the snapshot at *D*_em_ = 2). An increase in the strength of the electric field *D*_em_ results in the formation of percolating adsorbate clusters and their number increases with *D*_em_ (compare snapshots at *D*_em_ = 4, 6 and 8 in Figure 6a). Temporal evolution of the mean adsorbate concentration ⟨*x*⟩ and dispersion ⟨(δ*x*)^2^⟩ shown in Figure 6b indicates that patterns in Figure 6a are stationary ones. Moreover, it is seen that the electrical strength does not affect crucially both mean adsorbate concentration values and dispersion.

**Figure 7:**
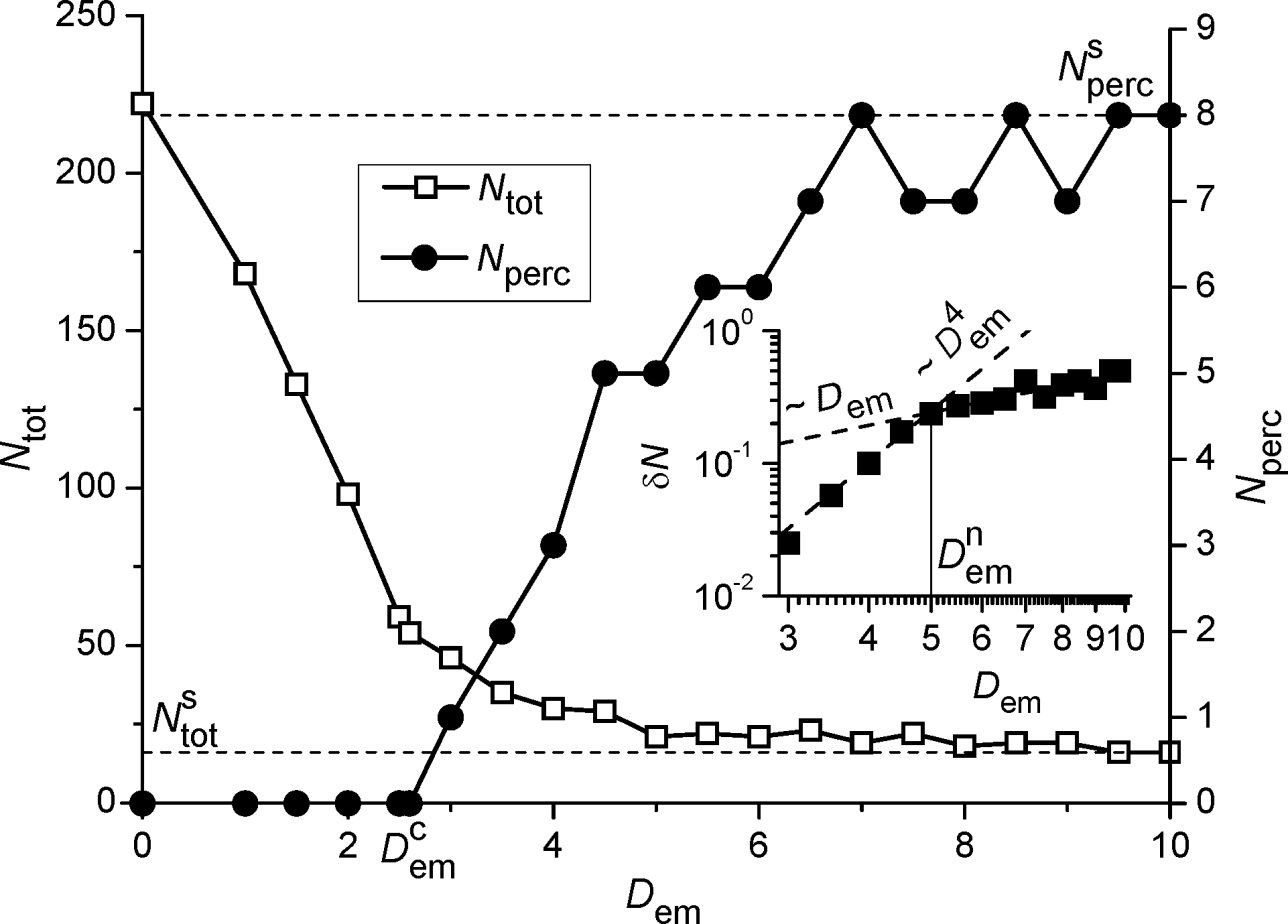
Dependencies of the total amount of adsorbate clusters *N*_tot_ and number of percolating clusters *N*_perc_ on the electrical field strength *D*_em_ at *k*_∥_ = 0.1 and α = 0.06. The inset shows a dependence of the ratio δ*N* = *N*_perc_/*N*_tot_ on the electrical field strength *D*_em_.

In Figure 7 we show the dependencies of the total amount of adsorbate clusters *N*_tot_ (empty squares) and number of percolating clusters *N*_perc_ (filled circles) on the electrical field strength *D*_em_. It is seen, that *N*_tot_ decreases with growing *D*_em_ and at elevated values of the electric field strength *N*_tot_ attains the value 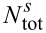 *>* 1. If *D*_em_ becomes larger, at some critical value 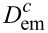
(α,*k*_∥_,ε,β) the number of percolating clusters *N*_perc_ starts to increase from zero and attains the value 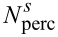. One can expect that at large *D*_em_ one will get 
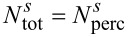
, when all adsorbate islands formed on the substrate will connect the opposite sides of the substrate. In the inset in Figure 7 we show the dependence of the ratio δ*N* = *N*_perc_/*N*_tot_ on the electrical field strength *D*_em_ on a log–log scale. It follows, that in the interval *D*_em_ ∈ (
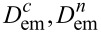
) one gets the rapid growth of the dependence δ*N*(*D*_em_) with the asymptote 
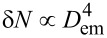
. This dynamics is realized due to separated elongated adsorbate clusters formed at *D*_em_ *<* 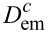 starting to combine into percolating clusters (see snapshots at *D*_em_ = 2 and *D*_em_ = 4 in Figure 6). At elevated *D*_em_ *>* 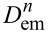 one gets a slow growth of the number of percolating adsorbate clusters (see filled circles in Figure 7) and asymptotic growth to δ*N* ∝ *D*_em_ occurs.

**Figure 8:**
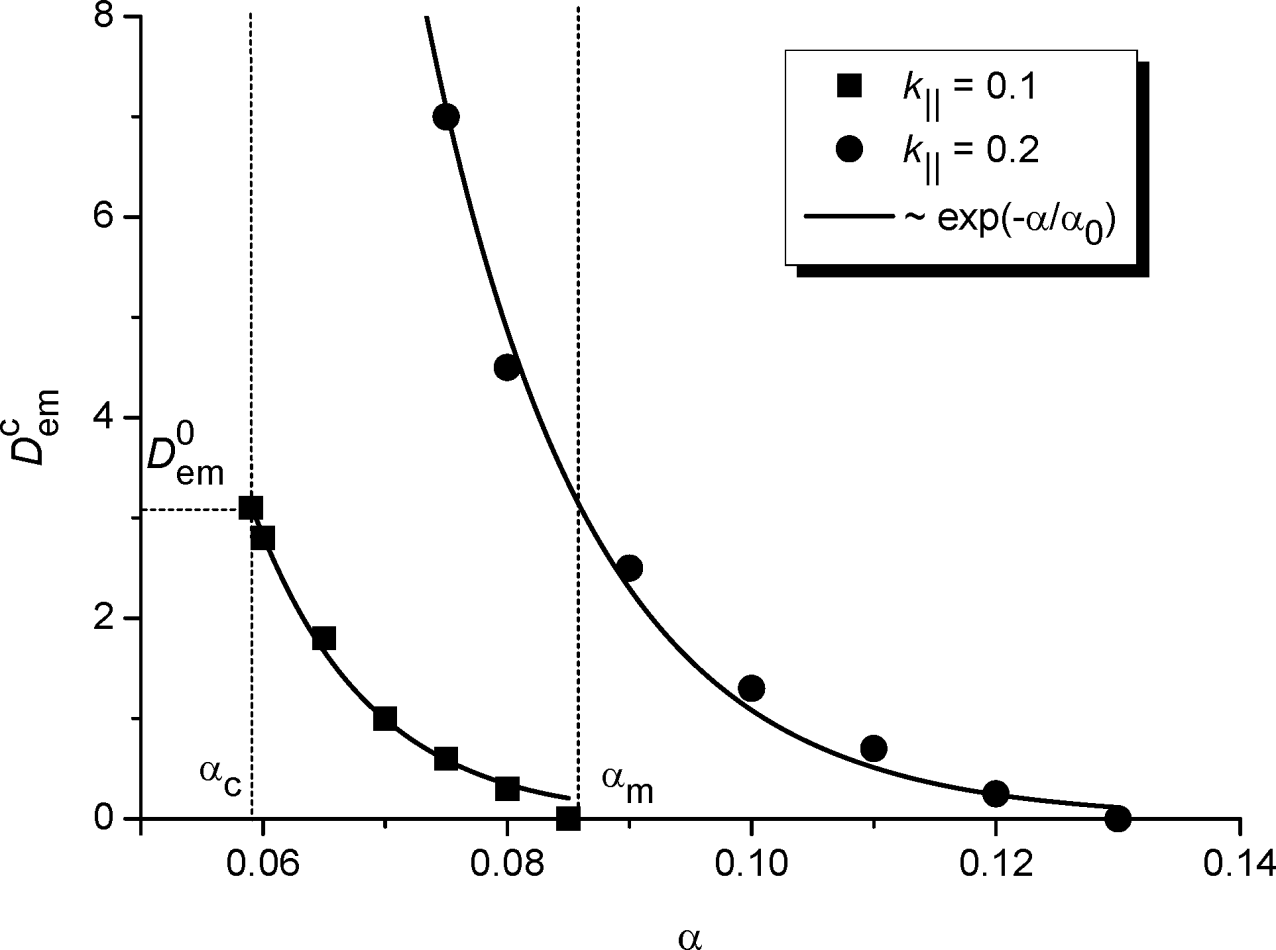
Dependence of the critical value of the electrical field strength 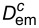 on the adsorption coefficient α at different values of the vertical diffusion coefficient *k*_∥_.

Next, let us discuss the dependence of the critical value of the electric field strength 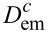, at which the percolating clusters of adsorbate begin to organize during deposition, on the adsorption coefficient α and the rate of the transference of adatoms between neighboring layers *k*_∥_. The corresponding dependencies 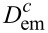
(α) at different *k*_∥_ are shown in Figure 8.

Let us initially discuss results for the case *k*_∥_ = 0.1, shown by filled squares in Figure 8. It follows that with an increase in the adsorption coefficient α from α_c_ to α_m_ (see Figure 4) the critical value of the strength of the applied electric field falls down to zero from the fixed value 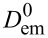
(*k*_∥_,ε,β). Hence, an increase in adsorption coefficient requires a smaller value of the strength of the applied electric field for the formation of percolating adsorbate clusters, which are realized above the dependence 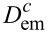
(α). It seems natural that an increase in α leads to a growth in the concentration of adsorbate on the substrate. In such a case the separated islands in an isotropic system become large and closely located. Therefore, at elevated α *<* α_m_ even a small strength of the applied electric force can induce the formation of percolating clusters.

An increase in the rate of adatoms transference between layers *k*_∥_ provides a decrease in adsorbate concentration on a substrate, and, as a result, requires elevated values of the strength of the applied electric force for the formation of percolating adsorbate clusters (see filled circles in Figure 8 at *k*_∥_ = 0.2). Obtained numerical data shown by filled symbols are fitted well by the exponential function exp(−α/α_0_) with the scaling parameter α_0_(*k*_∥_) (solid curves).

**Figure 9:**
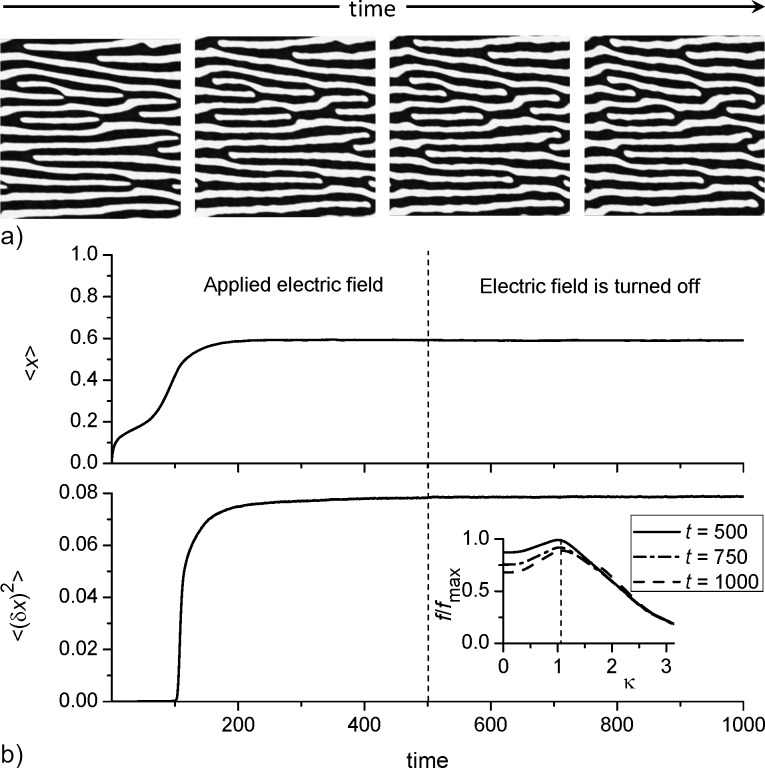
(a) Typical snapshots of the system evolution after the electric field is turned off at *k*_∥_ = 0.1, α = 0.06. The first snapshot corresponds to the case *D*_em_ = 6. (b) Evolution of the mean adsorbate concentration ⟨*x*⟩ (top panel) and the dispersion ⟨(δ*x*)^2^⟩ (bottom panel). The inset in Figure 9b shows the power spectral density function for the different times of deposition after the electric field is turned off.

Finally, let us discuss an important question from the applied physics perspective about the stability of the elongated morphology of the adsorbate islands when the electric field is turned off. To this end we fix *k*_∥_ = 0.1 and α = 0.06 and as initial conditions for the coverage field *x*(**r**) use the configuration obtained at *D*_em_ = 6 for *t* = 500 (see the corresponding snapshot in Figure 6a). Next, we solve the Equation 4 with the same values of the control parameters at *D*_em_ = 0. The obtained results are shown in Figure 9. From Figure 9a it follows, that after the electric field is turned off the morphology of the surface patterns (formed with applied electric field) does not change. The temporal dependencies of both the mean adsorbate concentration ⟨*x*⟩ and the dispersion ⟨(δ*x*)^2^⟩ (shown in Figure 9b with the applied electric field at *t <* 500 and without electric field at *t >* 500) illustrate that after the electric field is turned off both quantities dot not change in time. In the inset in the bottom panel in Figure 9b we show the power spectral density function *f*(κ) for the initial (*t* = 500), intermediate (*t* = 750) and final (*t* = 1000) stages rescaled by the maximal value *f*_max_ for the case *t* = 500. It follows, that the position of the peak of *f*(κ) does not change meaning that the period of spatial modulations of the coverage field *x*(**r**) remains constant. Hence, the elongated morphology of adsorbate islands remains stable if the electric field is turned off.

### Estimations

In order to test the parameter space used in our numerical simulations to offer a guidance for designing the corresponding experiments to observe patterns like the ones predicted by simulations we use the data from [78] for the system Ge on SiO_2_ at *T* = 773 K: activation energy for desorption *E*_d_ ≃ 0.44 eV and activation energy for diffusion *E*_D_ ≃ 0.24 eV. By exploiting the formula for the diffusion length *L*_D_ = 
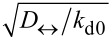
 with the lattice constant *a* = 5.6 × 10^−10^ for Ge we get *L*_D_ = *a*^2^exp((*E*_d_ − *E*_D_)/*T*) ≃ 50 nm. With the used relation *L* = 40*L*_D_, the mean linear size (diameter) of circular stationary adsorbate islands in the isotropic case with *D*_em_ = 0, shown in the top panel in Figure 3b, and the transverse size of elongated structures at *D*_em_ ≠ 0, shown in Figure 6a, are about 2*L*_D_ ≃ 100 nm. This is in good agreement with the experimental results discussed in [15,78].

## Conclusion

In this article we have studied the effects of electromigration on the morphology of growing thin films during condensation from the gaseous phase in the framework of numerical simulations. It is shown that an increase in the strength of the electrical field applied to the substrate results in the formation of elongated adsorbate islands in the direction of the electric field. If the field strength exceeds a critical value depending on the control parameters, then the adsorbate self-organizes into percolating islands on the substrate. We have shown that the critical value of the strength of the applied electrical field falls exponentially with the adsorption coefficient. An increase in the rate of adsorbate transfer between neighboring layers requires larger values of the electrical field strength for the formation of percolating islands on a substrate. It is shown that the elongated morphology of adsorbate islands remains stable if the electric field is turned off.

The general results obtained within this work can be used to predict the morphology of nanostructured thin films formed during condensation from the gaseous phase. The dimensionless control parameters of the model can be rescaled with the help of experimental data for any kind of system adsorbate/substrate to adjust the technological conditions for the growth of the nanostructured thin films with specified morphology and statistical characteristics.

## References

[1] Black J R (1969). IEEE Trans Electron Devices.

[2] Ho P S, Kwok T (1989). Rep Prog Phys.

[3] Tu K N (2003). J Appl Phys.

[4] Choi W J, Yeh E C C, Tu K N (2003). J Appl Phys.

[5] Chiang K N, Lee C C, Lee C C, Chen K M (2006). Appl Phys Lett.

[6] Chen C, Tong H M, Tu K N (2010). Annu Rev Mater Res.

[7] Tian T, Chen K, MacDowell A A, Parkinson D, Lai Y-S, Tu K N (2011). Scr Mater.

[8] Homma Y, Mcclelland R J, Hibino H (1990). Jpn J Appl Phys, Part 2.

[9] Williams E D, Fu E, Yang Y-N, Kandel D, Weeks J D (1995). Surf Sci.

[10] Gibbons B J, Noffsinger J, Pelz J P (2005). Surf Sci.

[11] Lin S-k, Liu Y-c, Chiu S-J, Liu Y-T, Lee H-Y (2017). Sci Rep.

[12] Leroy F, Karashanova D, Dufay M, Debierre J-M, Frisch T, Métois J-J, Müller P (2009). Surf Sci.

[13] Usov V, Coileain C O, Shvets I V (2010). Phys Rev B.

[14] Toktarbaiuly O, Usov V, Ó Coileáin C, Siewierska K, Krasnikov S, Norton E, Bozhko S I, Semenov V N, Chaika A N, Murphy B E (2018). Phys Rev B.

[15] Shklyaev A A, Latyshev A V (2019). Appl Surf Sci.

[16] Voigtländer B (2001). Surf Sci Rep.

[17] Shklyaev A A, Ichikawa M (2008). Phys-Usp.

[18] Shklyaev A A, Romanyuk K N, Kosolobov S S (2014). Surf Sci.

[19] Teys S A (2017). Appl Surf Sci.

[20] MacLeod J M, Lipton-Duffin J A, Lanke U, Urquhart S G, Rosei F (2009). Appl Phys Lett.

[21] Shklyaev A, Bolotov L, Poborchii V, Tada T (2015). J Appl Phys.

[22] Yongsunthon R, Tao C, Rous P, Williams E D, Michailov M (2011). Surface Electromigration and Current Crowding. Nanophenomena at Surfaces.

[23] Tao C, Cullen W G, Williams E D (2010). Science.

[24] Los Santos Valladares L D, Leon Felix L, Bustamante Dominguez A, Mitrelias T, Sfigakis F, Khondaker S I, Barnes C H W, Majima Y (2010). Nanotechnology.

[25] Gardinowski G, Schmeidel J, Pfnür H, Block T, Tegenkamp C (2006). Appl Phys Lett.

[26] Curiotto S, Müller P, El-Barraj A, Cheynis F, Pierre-Louis O, Leroy F (2019). Appl Surf Sci.

[27] Zhao J, Yu R, Dai S, Zhu J (2014). Surf Sci.

[28] Stoyanov S (1997). Surf Sci.

[29] Dufay M, Debierre J-M, Frisch T (2007). Phys Rev B.

[30] Chang J, Pierre-Louis O, Misbah C (2006). Phys Rev Lett.

[31] Pierre-Louis O (2006). Phys Rev Lett.

[32] Krug J, Dobbs H T (1994). Phys Rev Lett.

[33] Schimschak M, Krug J (1997). Phys Rev Lett.

[34] Du D, Srolovitz D (2004). Appl Phys Lett.

[35] Barakat F, Martens K, Pierre-Louis O (2012). Phys Rev Lett.

[36] Quah J, Margetis D (2010). Multiscale Model Simul.

[37] Leroy F, Müller P, Métois J J, Pierre-Louis O (2007). Phys Rev B.

[38] Chiu C-H, Huang Z, Poh C T (2006). Phys Rev B.

[39] Maroudas D (2011). Surf Sci Rep.

[40] Tomar V, Gungor M R, Maroudas D (2008). Phys Rev Lett.

[41] Khenner M (2013). C R Phys.

[42] Kim J H, Srolovitz D J, Cha P-R, Yoon J-K (2006). J Appl Phys.

[43] Du L, Maroudas D (2018). J Appl Phys.

[44] Kuhn P, Krug J, Hausser F, Voigt A (2005). Phys Rev Lett.

[45] Dasgupta D, Maroudas D (2013). Appl Phys Lett.

[46] Dasgupta D, Kumar A, Maroudas D (2018). Surf Sci.

[47] Kumar A, Dasgupta D, Dimitrakopoulos C, Maroudas D (2016). Appl Phys Lett.

[48] Solenov D, Velizhanin K A (2012). Phys Rev Lett.

[49] Casal S B, Wio H S, Mangioni S (2002). Phys A (Amsterdam, Neth).

[50] Kharchenko V O, Kharchenko D O (2012). Phys Rev E.

[51] Mangioni S E, Wio H S (2005). Phys Rev E.

[52] Mangioni S E (2010). Phys A (Amsterdam, Neth).

[53] Mikhailov A, Ertl G (1995). Chem Phys Lett.

[54] Battogtokh D, Hildebrand M, Krischer K, Mikhailov A S (1997). Phys Rep.

[55] Hildebrand M, Mikhailov A S, Ertl G (1998). Phys Rev Lett.

[56] Hildebrand M, Mikhailov A S, Ertl G (1998). Phys Rev E.

[57] Walgraef D (2003). Phys E (Amsterdam, Neth).

[58] Walgraef D (2004). Int J Quantum Chem.

[59] Kharchenko V O, Kharchenko D O, Kokhan S V, Vernyhora I V, Yanovsky V V (2012). Phys Scr.

[60] Kharchenko V O, Kharchenko D O, Dvornichenko A V (2014). Surf Sci.

[61] Walgraef D (2002). Phys E (Amsterdam, Neth).

[62] Kharchenko V O, Dvornichenko A V, Borysiuk V N (2018). Eur Phys J B.

[63] Kharchenko V O, Dvornichenko A V, Zhylenko T I (2020). Appl Nanosci.

[64] Dvornichenko A V, Kharchenko V O (2020). Phys Lett A.

[65] Kharchenko V O, Kharchenko D O, Dvornichenko A V (2016). Phys A (Amsterdam, Neth).

[66] Kharchenko D O, Kharchenko V O, Lysenko I O (2011). Phys Scr.

[67] Kharchenko D O, Kharchenko V O, Zhylenko T, Dvornichenko A V (2013). Eur Phys J B.

[68] Kharchenko V O, Kharchenko D O, Dvornichenko A V (2015). Eur Phys J B.

[69] Kharchenko V O, Kharchenko D O (2012). Eur Phys J B.

[70] Kharchenko V O, Kharchenko D O (2013). Condens Matter Phys.

[71] Kharchenko D O, Kharchenko V O, Bashtova A I (2014). Radiat Eff Defects Solids.

[72] Kharchenko V O, Kharchenko D O (2014). Phys Rev E.

[73] Kharchenko D O, Kharchenko V O, Bashtova A I (2013). Ukr J Phys.

[74] Sfyris G I, Gungor M R, Maroudas D (2010). Appl Phys Lett.

[75] Zauderer E (2006). Partial Differential Equations of Applied Mathematics.

[76] Grindrod P (1996). The Theory and Applications of Reaction-Diffusion Equations.

[77] Hundsdorfer W, Verwer J (2003). Numerical Solution of Time-Dependent Advection-Diffusion-Reaction Equations.

[78] Leonhardt D, Han S M (2009). Surf Sci.

